# User Perspectives on a Clinical Decision Tool to Support Individualized Exercise Prescriptions for Breast Cancer Survivors Not Meeting Exercise Guidelines: Cross-Sectional Survey

**DOI:** 10.2196/89463

**Published:** 2026-06-08

**Authors:** Emma Tian, Oliver WA Wilson, Jacob Schneider, Richard L Street, Jinani Jayasekera

**Affiliations:** 1 Division of Intramural Research National Institute on Minority Health and Health Disparities National Institutes of Health Bethesda, MD United States; 2 Department of Communication and Journalism Texas A&M University College Station, TX United States

**Keywords:** breast cancer, patients with breast cancer, physical activity, clinical decision support systems, exercise prescription, exercise discussion, exercise referral

## Abstract

**Background:**

More than 80% of breast cancer survivors do not meet the recommended levels of exercise, and <50% of health care providers promote exercise as part of survivorship care. Patient-provider communication may enhance exercise engagement by increasing patients’ understanding of exercise benefits and linking patients to resources, such as rehabilitation and exercise programs.

**Objective:**

This study aimed to explore perspectives on a novel clinical decision tool designed to support individualized exercise discussions and prescriptions among breast cancer survivors who do not meet exercise guidelines and health care providers who primarily treat such survivors.

**Methods:**

We conducted a cross-sectional online survey among US breast cancer survivors and health care providers. Participants were (1) female breast cancer survivors aged ≥35 years engaging in ≤150 minutes/week of moderate-intensity aerobic exercise or ≤2 days/week of muscle-strengthening exercise and (2) health care providers who had cared for breast cancer survivors within the past 12 months and reported below-average guideline adherence among their patients. Respondents reviewed a paper draft of a web-based clinical decision prototype tool for supporting individualized exercise discussions and prescriptions based on patients’ demographic, clinical, and contextual characteristics. We assessed perceived usefulness, potential uses (eg, counseling), preferred timing of access within clinical encounters, and preferences for tool characteristics (inputs/outputs).

**Results:**

The analytic sample comprised 26 breast cancer survivors and 69 health care providers. The survivors’ median age was 48 (IQR 37-65) years. Providers included patient navigators/social workers/nurses (29/69, 42.0%), breast oncologists (13/69, 18.8%), and occupational/physical therapists (12/69, 17.4%). The majority of providers (62/69, 89.9%, 95% CI 80.2%-95.8%) and survivors (23/26, 88.5%, 95% CI 69.8%-97.6%) reported that they would find the tool useful. Similarly, 85.5% of providers (59/69, 95% CI 75.0%-92.8%) and 84.6% of survivors (22/26, 95% CI 65.1%-95.6%) reported that the tool would increase their confidence to discuss exercise in a clinical setting. Both groups preferred that survivors access the tool with staff after a medical appointment (survivors: 20/26, 76.9%, 95% CI 56.4%-91.0%; providers: 58/67, 86.6%, 95% CI 76.0%-93.7%). Both groups also endorsed treatment history and readiness to exercise to consider as key inputs and improved quality of life and reduced treatment-related side effects as exercise benefits to communicate as tool outputs.

**Conclusions:**

The prototype tool concept was well received, with high endorsement of individual characteristics to consider and clinical benefits of exercise to communicate. Findings will inform refinement of the tool and future implementation testing in an understudied population of breast cancer survivors.

## Introduction

Breast cancer is the second-most common cancer among women in the United States, with an estimated four million survivors nationwide [1]. Advances in screening mammography and treatment have resulted in high survival rates [2]. However, breast cancer treatment may result in poor quality of life in cognition, sexual function, fatigue, and anxiety [3]. As a result, there is a need for interventions to improve health outcomes for survivors both postdiagnosis and during extended survivorship. Exercise, a low-risk lifestyle modification, could improve quality of life and reduce recurrence and mortality among breast cancer survivors [4-7]. However, national-level data indicate that only 13.3% of breast cancer survivors report participating in recommended levels of exercise [8].

Patient-provider interactions may provide opportunities to initiate conversations about exercise and facilitate exercise referrals for breast cancer survivors. Studies show that clinician discussions can significantly increase exercise participation among patients [9-12]. However, fewer than half of cancer survivors report receiving any exercise advice from their health care providers [13,14]. This gap may be attributed to barriers faced by both survivors and providers. Survivors may encounter persistent treatment-related side effects and fatigue as barriers to actual exercise participation [14,15], while providers often report lack of time, safety concerns, and a lack of knowledge on how or when to counsel patients about exercise [16,17].

Clinical decision tools may address these barriers by helping providers streamline and facilitate individualized conversations at the point of care by integrating medical knowledge with patient-specific data to inform decision-making [18]. For example, there are various clinical decision tools to guide treatment choices [19], such as the timing and selection of systemic therapy postdiagnosis [20,21]. However, there are currently no tools to support individualized exercise discussions and prescriptions for breast cancer survivors. To address this gap, we recently developed a prototype clinical decision tool to support patient-provider communication about exercise for breast cancer survivors [22].

Although both breast cancer survivors and health care providers could use the tool to engage in discussions about exercise, their perspectives and needs regarding this tool may differ. Understanding where patient-provider perspectives align and diverge is critical for informing necessary adjustments and, when appropriate, designing tailored interfaces for distinct user populations. Therefore, we collected survivors’ and providers’ perspectives on the tool to ensure its ability to address the needs and preferences of both stakeholders. Because this tool is likely to benefit survivors with low exercise adherence and the providers who care for these populations, our study focused on these groups. The primary aim of this study was to explore the perspectives of breast cancer survivors and health care providers on the characteristics of a prototype clinical decision tool for individualized exercise discussions and prescriptions.

## Methods

### Study Design and Tool Development

This study was a cross-sectional survey to explore breast cancer survivors’ and health care providers’ perspectives on a prototype clinical decision tool.

The Collaborative Deliberation Model, which posits five requirements for collaborative decision-making and behavior change (ie, constructive interpersonal engagement, alternative action recognition, learning, preference elicitation, and preference integration) [23,24], was used as a framework to develop the tool. Tool content was informed by the current literature and input from a multidisciplinary team of breast cancer survivors (n=5), exercise specialists (n=4), physicians (n=3), and a tool developer (n=1).

The tool was designed to generate individualized health outcomes (eg, projected life-years) associated with varying levels (ie, amounts and types) of exercise (eg, 150 minutes/week of moderate-intensity aerobic exercise) using a validated microsimulation model [25,26]. Results from the microsimulation model are available online [27]. To build the model [28], we adapted an established simulation modeling approach developed within the Cancer Intervention and Surveillance Modeling Network (CISNET) to estimate breast cancer–specific survival, all-cause survival, and life-years associated with different levels of postdiagnosis exercise among 50-75-year-old (postmenopausal) women diagnosed with stage I-III invasive breast cancer. Model estimates were generated for over 60,000 combinations of simulated individual (age, weight status) and clinical (stage, tumor subtype) characteristics, breast cancer treatment (eg, hormonal therapy, chemotherapy), and different levels of exercise (eg, 30 minutes/week of moderate-intensity aerobic exercise). The model was validated using independent data from the Women’s Healthy Eating and Living Study [29]. The model results showed that survival rates and absolute benefits for exercise vary by age, weight status, stage, tumor subtype, and the amount and type of exercise. For example, for a 65-69-year-old woman with obesity and stage II, hormone receptor–positive, human epidermal growth factor receptor-2–negative breast cancer, the model estimated 10-year breast cancer–specific and all-cause survival associated with no/minimal exercise to be 79.2% and 72.2%, respectively. Increasing aerobic exercise from none/minimal to insufficient was associated with absolute increases of 2.8% and 3.4% for 10-year breast cancer–specific and all-cause survival, respectively. The model closely replicated survival rates in independent data.

**Figure 1:**
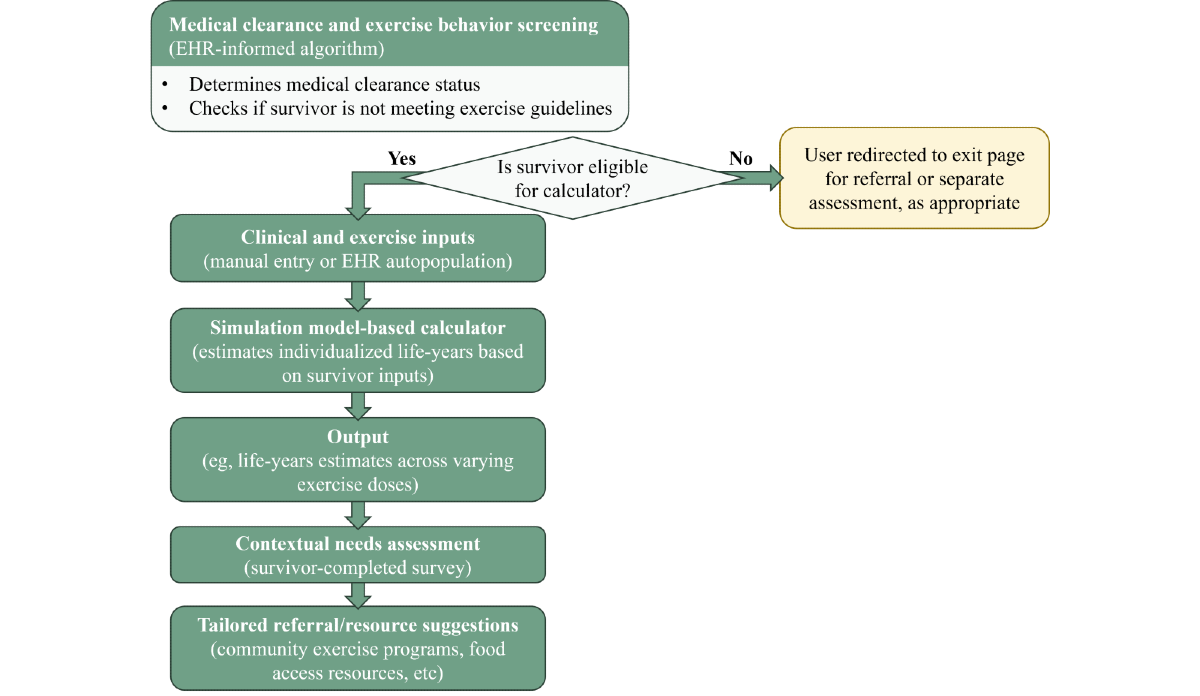
Workflow of the clinical decision tool to support exercise among breast cancer survivors. EHR: electronic health record.

The model logic and clinical workflow for the tool prototype are illustrated in Figure 1. Prior to calculator use, survivors are screened for eligibility (ie, medically cleared to exercise and currently not meeting exercise guidelines). For eligible users, model inputs include individual demographic (eg, age) and clinical (eg, cancer stage at diagnosis and treatment history) characteristics obtained from the electronic health record, when available, or entered by clinical staff or the patient. Using these inputs, the tool calculates individualized estimates of health outcomes across different levels of exercise for providers to discuss the benefits of exercise with patients. In addition, contextual factors are collected and used to tailor exercise referral and resource recommendations (eg, nearby gym or group exercise classes). In this study, the paper draft of the prototype tool shown to participants depicted projected life-years associated with varying exercise levels, allowing them to estimate average life-year gains from increased exercise using the model output. All participants reviewed the same prototype output.

### Survey Development and Refinement of Tool Prototype

We developed two surveys using items selected from existing instruments and prior studies (Table S1 in Multimedia Appendix 1). Sociodemographic items were drawn from the National Institutes of Health (NIH) Common Data Elements repository [30]. Items related to potential tool inputs and outputs were informed by prior studies [31] and cancer exercise guidelines [32]. Items were adapted for the survey and scored using a 5‑point Likert scale (1=strongly disagree to 5=strongly agree) to assess participants’ endorsement. A Spanish-language version of the survivor survey was developed with input from two Spanish-speaking researchers via forward-backward translation. Before deploying the survey, cognitive interviews were conducted with 5 survivors (n=1, 20%, Spanish speaking and n=4, 80%, English speaking) and 10 health care providers to evaluate their comprehension of the tool and survey and to gather feedback on items to add or revise. For instance, participants suggested incorporating additional input characteristics into the tool (eg, exercise readiness).

### Participants

A third-party contractor recruited breast cancer survivors and health care providers to complete the online survey evaluating the prototype clinical decision tool.

#### Breast Cancer Survivors

Survivors were recruited using a patient panel. Inclusion criteria were (1) female breast cancer survivor, (2) aged ≥35 years, (3) residing in the United States, and (4) not meeting exercise guidelines (defined as self-reporting ≤150 minutes/week of moderate-intensity aerobic exercise or ≤2 days/week of muscle-strengthening exercise) [32]. Cognitive interview participants (n=5) were excluded from the survey sample. Duplicate responses were prevented by allowing only one response per unique panel identifier.

#### Health Care Providers

Providers were recruited through professional organizations (eg, American Society of Clinical Oncology), publicly available information, and convenience and snowball sampling. Inclusion criteria were (1) health care professional (exercise specialist, nurse, occupational/physical therapist, patient navigator, physician [breast oncologist and primary care provider], and social worker), (2) having treated or provided care to at least one breast cancer survivor aged ≥35 years within the past 12 months (June 2023-June 2024), and (3) serving a patient population with lower exercise participation rates than reported in the general breast cancer survivor population [8]. Specifically, providers separately reported the percentage of their breast cancer survivor patients meeting aerobic and muscle-strengthening exercise guidelines. Providers were eligible if ≤40% of their patients met aerobic or ≤20% met muscle-strengthening exercise guidelines. Cognitive interview participants (n=10) were excluded from the survey sample. Duplicate responses were monitored by reviewing IP addresses, although multiple providers from the same institution (eg, same hospital) sharing an IP address were permitted.

### Data Collection

Screening questions confirmed respondents’ eligibility prior to survey access. To enhance data quality, responses were reviewed for straight-lining and excessively short completion times. Responses were required to meet a completeness threshold of 80% of survey items for inclusion in analyses. Data were collected in June 2024. The sample size was determined based on practical considerations, including time and resources [33].

### Survey Measures

#### Participants’ Sociodemographic Characteristics

Both survivors and providers reported their age [30], race [30], ethnicity [30], and state and urbanicity (urban vs rural [34]) of US residence/practice. Survivors also reported their education [30], income [30], employment status [30], and survivorship stage [35], while providers reported their profession [35] and proportion of patients treated in each survivorship stage.

#### Participants’ Perspectives on a Clinical Decision Tool for Individualized Exercise Discussions and Prescriptions

Participants were presented with a paper draft of the prototype tool (Figure S1 in Multimedia Appendix 1) and asked to rate various aspects using a 5-point Likert scale. They reported their perceptions of the tool’s usefulness, their likelihood of regular use, and whether it would increase their confidence in discussing exercise in a clinical context. In addition, they provided input on the optimal timing for tool access (eg, before consultation) [31]. Participants who reported that they would find the tool useful in clinical settings further rated its functionalities. They indicated their level of agreement that the tool could educate on exercise benefits and harms, encourage exercise engagement, facilitate shared decision-making, identify exercise resources, and refer patients to exercise professionals. Participants rated their agreement with the inputs used to generate individualized exercise estimates and determine medical clearance (eg, adverse events associated with breast cancer and its treatment). They also rated the outputs intended to communicate exercise benefits (eg, improved bone health). Participants were also given an opportunity to provide feedback through open-ended questions.

### Statistical Analyses

Categorical variables were presented as frequencies and percentages, while continuous variables were summarized using medians (IQRs). For our primary aim, we examined within-group percentage endorsement of tool characteristics among survivors and providers to inform design prioritization. Responses using the 5-point Likert scale were dichotomized as endorsement (agree or strongly agree) versus nonendorsement (neutral, disagree, and strongly disagree) [31]. We used these dichotomized variables in our primary analysis to assess whether each tool characteristic met a practical threshold of favorable support. In this context, measures of central tendency (eg, mean and median) were less useful to inform prioritization because responses tended to cluster at the upper end of the scale, making small differences in mean or median scores less directly interpretable. Percentage endorsement and 95% CIs were calculated for each group using the exact Clopper-Pearson method [36]. For sensitivity analysis, we also reported the mean (SD) of Likert ratings (1-5) for each item. Due to differences in sampling strategies, inclusion criteria, and underlying population characteristics between the two groups, we did not perform direct comparisons between survivors and providers but described patterns of endorsement within categories of tool attributes for each group. Participants with missing data were included, and the number of respondents for each question was reported. Open-text responses to a question about how the tool would increase confidence to discuss exercise in clinical settings were analyzed using thematic analysis [37]. All analyses were performed using Stata (version 18.0).

### Ethical Considerations

This study was approved by the Health Media Lab Institutional Review Board (IRB #00001211, IORG #0000850, FWA #00001102; protocol #2470). The NIH IRB determined that NIH investigators’ use of the deidentified dataset provided by the external contractor was exempt. Informed consent was obtained from all participants prior to survey administration. Data were deidentified before analysis to protect participant privacy and confidentiality. Each survivor received US $75 for cognitive interviews and US $30 for completing the survey, while each provider received ≥US $125 for cognitive interviews and ≥US $60 for completing the survey.

## Results

### Participants’ Characteristics

Table 1 presents the sociodemographic characteristics of breast cancer survivors and health care providers.

Of 142 women screened, complete survey responses were collected from 26 (18.3%) breast cancer survivors (Figure S2 in Multimedia Appendix 1). The survivors’ median age was 48 (IQR 37-65) years. Self-identified races/ethnicities were Hispanic (n=7, 26.9%), non-Hispanic White (n=6, 23.1%), American Indian/Alaska Native (n=5,19.2%), Asian (n=4, 15.4%), non-Hispanic Black (n=2, 7.7%), and Middle Eastern/North African (n=2, 7.7%). In addition, 65.4% (n=17) survivors were in the posttreatment stage, 46.2% (n=12) resided in the Southern United States, 76.0% (n=20) had a college degree, and 62.5% (n=16) reported an annual household income ≥US $75,000 (Table S2 in Multimedia Appendix 1).

**Table 1:** Characteristics of breast cancer survivors and health care providers.

Characteristics			Breast cancer survivors (n=26)		Health care providers (n=69)
Age (years), median (IQR)			48 (37-65)		37 (31-47)
**Gender, n (%)**					
	Female	26 (100.0)		61 (88.4)	
	Male	0		8 (11.6)	
**Race and/or ethnicity, n (%)**					
	Asian	4 (15.4)		8 (11.6)	
	American Indian/Alaska Native	5 (19.2)		0 (0.0)	
	Non-Hispanic Black	2 (7.7)		3 (4.4)	
	Hispanic	7 (26.9)		1 (1.5)	
	Middle Eastern and North African	2 (7.7)		0	
	Non-Hispanic White	6 (23.1)		52 (75.4)	
	Mixed	0		4 (5.8)	
	Other	0		1 (1.5)	
**Survivorship stage, n (%)/median% (IQR%)**					
	Pretreatment	0		5 (0-10)^a^	
	On treatment	8 (30.8)		40 (20-55)^a^	
	Posttreatment	17 (65.4)		30 (10-50)^a^	
	Palliative care	1 (3.9)		5 (0-10)^a^	
**Region, n (%)**					
	Midwest	3 (11.5)		7 (10.1)	
	Northeast	4 (15.4)		23 (33.3)	
	South	12 (46.2)		18 (26.1)	
	West	7 (26.9)		21 (30.4)	
**Urbanicity/rurality, n (%)**					
	Rural	4 (15.4)		5 (7.3)	
	Suburban	12 (46.2)		28 (40.6)	
	Urban	10 (38.5)		36 (52.2)	

^a^For providers, the survivorship stage indicated the percentage of patients treated at each stage of survivorship. Values are presented as the percentage median (IQR).

Of 254 health care providers screened, complete survey responses were collected from 69 (27.2%) providers (Figure S2 in Multimedia Appendix 1). The providers’ median age was 37 (IQR 31-47) years, and 88.4% (n=61) were female. The group comprised 29 (42.0%) patient navigators/social workers/nurses, 13 (18.8%) breast oncologists, 12 (17.4%) occupational/physical therapists, 11 (15.9%) exercise specialists, and 4 (5.8%) primary care physicians (Table S2 in Multimedia Appendix 1). The group was predominantly non-Hispanic White (n=52, 75.4%), and the most common practice region was the Northeastern United States (n=23, 33.3%). Providers reported that a median of 40% (IQR 20%-55%) of their patients were currently receiving treatment.

### Findings

#### Tool Usefulness, Potential Uses, and Timing

Table 2 summarizes the within-group percentage endorsement among breast cancer survivors and health care providers regarding perceived usefulness of the tool, potential uses, and preferred timing of access. Over 80% of both groups endorsed that they would find the tool useful (survivors: 23/26, 88.5%, 95% CI 69.8%-97.6%; providers: 62/69, 89.9%, 95% CI 80.2%-95.8%).

**Table 2:** Endorsement by breast cancer survivors and health care providers of the usefulness, potential uses, and timing of access of the clinical decision tool to support exercise among survivors.^a^

Questions		Breast cancer survivors, n/N (%); 95% CI (%)		Health care providers, n/N (%); 95% CI (%)
**Usefulness**				
	Would increase your self-confidence to talk about exercise with your health care provider(s)/patients	22/26 (84.6); 65.1-95.6	59/69 (85.5); 75.0-92.8	
	You would find useful	23/26 (88.5); 69.8-97.6	62/69 (89.9); 80.2-95.8	
	You would use on a regular basis	20/26 (76.9); 56.4-91.0	53/69 (76.8); 65.1-86.1	
**Uses**				
	Encourage to engage in exercise	23/23 (100.0); 85.2-100	60/62 (96.8); 88.8-99.6	
	Educate on the benefits (and harms) of engaging in exercise	21/23 (91.3); 72.0-98.9	58/62 (93.5); 84.3-98.2	
	Refer to exercise professionals	20/23 (90.9); 70.8-98.9	51/62 (83.6); 71.9-91.8	
	Facilitate shared decision-making between patient and provider about engaging in exercise	21/23 (91.3); 72.0-98.9	57/62 (91.9); 82.2-97.3	
	Identify resources available within the health care system to support engagement in exercise	21/23 (91.3); 72.0-98.9	55/62 (88.7); 78.1-95.3	
**Timing of access to the tool for survivors**				
	Before a medical appointment (individually)	19/26 (73.1); 52.2-88.4	43/69 (62.3); 49.8-73.7	
	Before a medical appointment (with a care coordinator or patient navigator)	17/26 (65.4); 44.3-82.8	41/68 (60.3); 47.7-72.0	
	During a medical appointment with a health care provider	20/26 (76.9); 56.4-91.0	55/68 (80.9); 69.5-89.4	
	After a medical appointment (individually)	18/26 (69.2); 48.2-85.7	57/66 (86.4); 75.7-93.6	
	After a medical appointment (with a care coordinator or patient navigator)	20/26 (76.9); 56.4-91.0	58/67 (86.6); 76.0-93.7	

^a^Responses on the 5-point Likert scale (strongly disagree to strongly agree) were dichotomized as endorsement (agree/strongly agree) vs nonendorsement. Percentages were calculated using the number of respondents who provided a valid response to each question. Due to missing data, denominators differed across items, and the same “n” was associated with different percentages.

Regarding potential uses, the top-ranked uses were similar for both groups: encouragement to engage in exercise (survivors: 23/23, 100.0%, 95% CI 85.2%-100.0%; providers: 60/62, 96.8%, 95% CI 88.8%-99.6%), education on the benefits and harms of exercise (survivors: 20/23, 91.3%, 95% CI 72.0%-98.9%; providers: 58/62, 93.5%, 95% CI 84.3%-98.2%), and facilitating shared decision-making between patients and providers about exercise (survivors: 21/23, 91.3%, 95% CI 72.0%-98.9%; providers: 57/62, 91.9%, 95% CI 82.2%-97.3%).

Analysis of responses to the open-ended question about how the tool could increase confidence to discuss exercise in clinical settings identified several themes (Table S3 in Multimedia Appendix 1). Among providers, the main themes included access to evidence-based information, guidance to discuss exercise with patients, and increased knowledge. For instance, one provider noted:

It would give me evidence-based guidance on how to discuss important exercise topics to my patients.

Clinical exercise physiologist, urban cancer center

Similarly, among survivors, key themes included access to evidence to take to their provider, exercise knowledge, and support to prepare for and initiate conversations about exercise with a provider. For instance, one survivor explained:

I would have specific exercise knowledge and confidence to talk to a professional about routine exercises.

70-year-old survivor, posttreatment stage

In terms of the preferred timing for survivors to access the tool, survivors favored accessing the tool with a provider during a medical appointment or with a care coordinator/patient navigator postappointment (both 20/26, 76.9%, 95% CI 56.4%-91.0%), followed by individually preappointment (19/26, 73.1%, 95% CI 52.2%-88.4%). Similarly, providers preferred that survivors access the tool postappointment with a care coordinator/patient navigator (58/67, 86.6%, 95% CI 76.0%-93.7%) or individually (57/66, 86.4%, 95% CI 75.7%-93.6%) and during an appointment (55/68, 80.9%, 95% CI 69.5%-89.4%). Relative to other timing options, providers showed lower endorsement for survivors to access the tool preappointment.

Table S4 in Multimedia Appendix 1 provides full Likert distributions and mean (SD) scores for perceived usefulness of the tool, potential uses, and preferred timing of access. Overall, the highest proportion of respondents strongly agreed that they would find the tool useful and that they would use the tool for encouragement to engage in exercise, provide referral to exercise professionals, and facilitate shared decision-making about exercise. Relative to other timing options for tool access (ie, during or after a medical appointment), a higher proportion of providers selected “neutral” or “disagree” regarding survivor access to the tool before the appointment, either individually (neutral: 12/69, 17.4%; disagree: 12/69, 17.4%) or with medical staff (neutral: 14/68, 20.6%; disagree: 12/68, 17.6%).

#### Tool Features With the Highest Endorsement Among Participants

Table S5 in Multimedia Appendix 1 lists the tool feature items with the highest endorsement for inclusion by group. These items encompassed inputs (clinical data, health and exercise information, contextual factors, adverse events) and outputs (benefits). Table S6 and Table S7 in Multimedia Appendix 1 present within-group endorsement percentages and mean (SD) Likert scores, respectively, for all tool features. Within-group rank-order sensitivity analyses using mean Likert scores yielded patterns broadly consistent with the dichotomized endorsement analyses.

#### Clinical Inputs

Current treatment information was the highest-rated clinical input among both groups (survivors: 22/26, 84.6%, 95% CI 65.1%-95.6%; providers: 65/69, 94.2%, 95% CI 85.8%-98.4%). Historical treatment was highly endorsed by both groups (survivors: 21/26, 80.8%, 95% CI 60.6%-93.4%; providers: 57/69, 82.6%, 95% CI 71.6%-90.7%). In addition, tumor characteristics were supported by survivors (20/26, 76.9%, 95% CI 56.4%-91.0%), while providers strongly endorsed age (62/69, 89.9%, 95% CI 80.2%-95.8%).

#### Overall Health and Exercise Inputs

Providers showed exceptionally high endorsement across all overall health and exercise inputs (range 95.6%-100.0%). For instance, 100% (69/69, 95% CI 94.8%-100.0%) of providers endorsed the inclusion of various impairments (functional, physical, cognitive). Among survivors, the highest endorsement was for readiness to exercise (22/26, 84.6%, 95% CI 65.1%-95.6%), followed by current comorbidities (20/25, 80.0%, 95% CI 59.3%-93.2%) and cognitive impairment (20/26, 76.9%, 95% CI 56.4%-91.0%).

#### Contextual Inputs

In both groups, access to exercise resources at home was the top-rated contextual input (survivors: 24/26, 92.3%, 95% CI 74.9%-99.1%; providers: 64/69, 92.8%, 95% CI 83.9%-97.6%). Access to healthy food was also highly endorsed by both groups (survivors: 22/26, 84.6%, 95% CI 65.1%-95.6%; providers: 60/69, 87.0%, 95% CI 76.7%-93.9%).

#### Adverse Event Inputs

Slowing and fatigue emerged as the top-rated adverse event inputs for both groups (survivors: 24/26, 92.3%, 95% CI 74.9%-99.1%; providers: 66/67, 98.5%, 95% CI 92.0%-100.0%). Among survivors, sarcopenia and neuropathy shared the highest rating with slowing and fatigue. Among providers, the next most highly endorsed inputs were lymphedema (67/69, 97.1%, 95% CI 89.9%-99.6%) and bone loss (66/69, 95.7%, 95% CI 87.8%-99.1%).

#### Outputs (Benefits of Exercise)

Among survivors, the top-rated benefits were fewer treatment side effects and reduced cardiovascular death risk (24/26, 92.3%, 95% CI 74.9%-99.1%), followed by improved life expectancy (23/25, 92.0%, 95% CI 74.0%-99.0%) and improved bone health (23/26, 88.5%, 95% CI 69.8%-97.6%). Providers highly endorsed improved quality of life (69/69, 100.0%, 95% CI 94.8%-100.0%), along with improved bone health and a greater ability to perform everyday tasks (67/68, 98.5%, 95% CI 92.1%-100.0%). Providers also strongly endorsed fewer depressive symptoms (67/69, 97.1%, 95% CI 89.9%-99.6%).

## Discussion

### Principal Findings

Studies demonstrate the utility of clinical decision tools in addressing complex health care decisions [20,38,39]. However, there are limited data on the involvement of users in the design process or on their specific decision support needs. Studies on existing tools for breast cancer surgery and treatment provide some insights into the user experience (eg, general usability information) but lack quantitative data on the specific decision-making needs and priorities of patients and providers [19,40]. To the best of our knowledge, this study is the first to quantitatively evaluate the criteria prioritized by breast cancer survivors who do not meet exercise guidelines and health care providers treating populations with low exercise adherence. Understanding the needs of this population is important because survivors not meeting recommended levels of exercise stand to benefit the most from increasing exercise to guideline levels. This study’s insights will help not only guide the tool’s design and implementation but also enhance the understanding of broader exercise decision support needs among health care providers and breast cancer survivors.

Our study explored the preferred timing for survivors to access the tool during their medical visit. In addition to accessing the tool postappointment with a care coordinator or patient navigator, survivors also favored accessing the tool individually preappointment. However, providers showed lower endorsement for this timing, which may reflect concerns about patients initiating exercise without medical clearance [41]. Prior research indicates that providing patients with direct advice and access to information outside of clinical encounters improves outcomes and aligns with patients’ preferences to access information on their own time [42,43]. Thus, a mutually acceptable strategy may involve survivors accessing the tool before and/or after a medical appointment with a care coordinator or patient navigator.

An alternative timing of tool deployment could support specific aspects of effective collaborative decision-making. First, effective decision-making requires patients to be informed about their condition and treatment [44]. Therefore, this tool could serve as a preconsultation aid [45], providing information about exercise options and clarifying what best fits the patient’s preferences and needs [46]. Second, effective decision-making requires patients to actively discuss their preferences and needs with provider support [44]. During the consultation, the tool could foster collaborative goal setting with the clinician, resulting in an action plan for the patient [47].

Over 80% of providers and survivors endorsed that they would find the tool useful and that it would increase their confidence to discuss exercise in a clinical setting. To help optimize the tool, we identified high-priority items that both survivors and providers agreed should be considered in exercise discussions. There was overlap between both groups in the top-rated inputs for calculating exercise estimates. For instance, historical and current treatments were highly prioritized by both groups, consistent with the prior literature on their influence on health and exercise [48,49]. In terms of the top outputs (benefits), both survivors and providers highly prioritized information related to improved quality of life and greater ability to perform everyday tasks. This is consistent with the prior literature on breast cancer survivors’ health concerns [50]. Given the evidence that exercise improves quality of life and functional ability [51,52], we plan to include treatment-related side effects, sleep, and cognitive abilities in the tool. It is also noteworthy that, among survivors, improved life expectancy was highly prioritized, even more so than breast cancer or all-cause death risk. This suggests that survival information with positive framing (eg, gains vs losses) may be more appealing and potentially serve as a motivator for survivors to exercise. Such findings align with prior studies indicating that gain-framed messages may be more effective for preventive behaviors, such as exercising [53,54]. However, lower cardiovascular death risk was the top priority for survivors. This may reflect concerns about the cardiovascular risks associated with cancer treatments [55,56]. To address these concerns, we plan to model cardiovascular death risk in the tool. Fatigue, a common concern in breast cancer treatment, was one of the top adverse events identified by both groups. Extensive literature documents the prevalence, impact, and beneficial effects of exercise [57-61]. We plan to integrate estimates of exercise’s impact on fatigue into our tool to support informed decision-making.

Contextual factors (eg, access to healthy food, exercise resources) may contribute to racial, ethnic, and socioeconomic disparities in exercise participation and health outcomes (eg, survival, quality of life) among breast cancer survivors in the United States [62-66]. Therefore, our tool will incorporate contextual factors to support tailored exercise recommendations. For example, information about local exercise programs (eg, LIVESTRONG) could facilitate recommendations, while information about access to exercise resources could guide suggestions for home‑based exercises or low‑cost equipment. In real‑world implementation, these data would ideally be collected through existing clinical workflows (eg, standardized screenings used in primary care or patient‑reported intake forms), although in some settings, additional data collection may be required. To minimize burden, contextual data would be optional and collected using nonstigmatizing language, with missingness permitted. To mitigate disparities in tool use, the tool would remain functional even when contextual data are incomplete. For instance, information about home‑based exercises, healthy food options, and local resources will be provided without individual‑level data.

### Strengths and Limitations

Our study had several strengths. First, applying user-centered design principles with an iterative process helped tailor the tool to meet the specific needs and preferences of its intended users [67,68]. In addition, we addressed a gap in the literature by surveying breast cancer survivors not meeting exercise guidelines. Although studies have identified that information about exercise is an unmet need among approximately 40% of breast cancer survivors [69,70], there are limited data on the specific exercise information and considerations relevant to breast cancer survivors not meeting exercise guidelines. Our study helps address this gap. Furthermore, our health care provider sample included a range of professions involved in delivering survivorship care. Input from breast cancer survivors and health care providers will be incorporated into an online version of the tool for further testing and implementation of the tool in real-world settings.

As an exploratory study, our findings should be interpreted considering several limitations. First, the modest sample size and convenience sampling used in this study may limit the generalizability of our findings. For instance, the breast cancer survivors in our sample were younger than the median age at breast cancer diagnosis (median 48 vs 62 years) [2,71] and had higher levels of education and household income than national estimates [8]. Younger survivors may be more comfortable with digital tools and less burdened by comorbidities. Furthermore, the online format likely excluded individuals without internet access [72,73]. Therefore, endorsement of the tool may have been overestimated in our sample relative to the broader population of breast cancer survivors, particularly among older survivors or those with lower digital access or health literacy. Second, this study evaluated perceptions of a paper-based prototype rather than a functional tool. Although paper-based prototyping is a well-established low-cost and efficient method in tool development [74-76], our findings may have been susceptible to hypothetical bias [77]. The literature comparing self-reported and revealed preferences indicates that individuals tend to overstate their valuation of services, particularly in hypothetical settings [78-80]. Accordingly, self-reported preferences in this study may not be generalizable to real-world use or translate into behavioral changes in practice. Nevertheless, our approach in this study was appropriate for an early-stage, user-centered design study, where the primary goal was to gather initial feedback on content, structure, and perceived usefulness prior to developing a functional tool [40]. To help mitigate potential biases, we encouraged participants to provide candid feedback in open-ended questions. In a future study, a fully functional version of the tool incorporating health care provider and patient perspectives from this study will be used to assess the impact of the tool on behavioral change and clinical outcomes.

### Conclusion

Our findings suggest considerable concordance between breast cancer survivors’ and health care providers’ perceptions of priorities and informational needs for an individualized exercise decision tool. Both groups prioritized information about quality of life, the ability to perform everyday tasks, and fatigue, aligning with prior qualitative literature [81-83]. These findings provide formative, user-centered insights to inform the continued development of an individualized clinical decision tool to support patient-provider communication about exercise. Because this study did not evaluate a functional tool, our findings reflect perceived usefulness and acceptability rather than actual usability or changes in communication, referral behavior, or exercise uptake. Future research will involve real-world usability testing and evaluation of the tool’s impact on communication, referral practices, and exercise-related outcomes.

## Appendix

Multimedia Appendix 1 Survey question sources; breast cancer survivor and health care provider characteristics; thematic analysis of respondents' open-ended responses regarding how the tool would increase their confidence to discuss exercise in clinical settings; Likert scale distributions of breast cancer survivors’ and health care providers’ endorsement of usefulness, potential uses, and timing of access to the tool; survey items with the highest endorsement among breast cancer survivors and health care providers; breast cancer survivors’ and health care providers’ endorsement of inputs and outputs for inclusion in an exercise clinical decision tool; mean (SD) of Likert scale (1=strongly disagree to 5=strongly agree) responses among breast cancer survivors and health care providers; screenshot of the paper draft of the prototype clinical decision tool for individualized exercise discussions shown to study participants; and flow diagram of study participants (breast cancer survivors and health care providers).

## Data Availability

The datasets generated and analyzed during the study are available from the corresponding author upon reasonable request.
