# Supplementary material for: User Perspectives on a Clinical Decision Tool to Support Individualized Exercise Prescriptions for Breast Cancer Survivors Not Meeting Exercise Guidelines: Cross-Sectional Survey
Source: JMIR Form Res. 2026 Jun 8;10:e89463. doi: 10.2196/89463 (PMC13287976; doi:10.2196/89463)
Supplement: Multimedia Appendix 1 [file formative_v10i1e89463_app1.docx]

**Table S1.** Survey question sources.

| **Measure** | **Breast cancer survivor** | **Health care provider** | **Source** |
| --- | --- | --- | --- |
| **Survey participants’ characteristics** | | | |
| Sociodemographic | Age, race, ethnicity, state | | NIH CDE repository [1]  and Powers et al. [2] |
|  | Urbanicity/rurality of residence | Urbanicity/rurality of practice |  |
|  | Household income | - |  |
|  | Education level | - |  |
| Clinical | Survivorship stage | Proportion of patients in each survivorship stage | Cancer exercise guidelines [3], Clinicians’  Perspectives on  Exercise in Patients  with Cancer (CliPEC)  questionnaire [4], and cognitive interview |
|  | - | Health care profession |  |
| **Tool characteristics and functionalities** | | | |
| Timing | Before a clinical consultation (on their own); before a clinical consultation (with a care coordinator or patient navigator); during a clinical consultation with the health care provider; after clinical consultation (on their own); after a clinical consultation (with a care coordinator or patient navigator) | | O’Neill et al. [5] |
| Tool Inputs | **Demographic and clinical:** age, race and ethnicity, stage at diagnosis, tumor characteristics, current treatment (e.g., chemotherapy, radiotherapy, endocrine therapy, surgery), and historical treatment | | NIH CDE repository [1], cancer exercise guidelines [3], and cognitive interview |
|  | **Overall health and exercise:** current comorbidities, physical impairments (e.g., mobility, reaching, etc.), functional impairments (e.g., challenges with daily living), cognitive impairments (e.g., changes in memory or attention, difficulty problem solving, etc.), psychological impairments (e.g., changes in mood, feeling down, etc.), patient personal preferences (e.g., what activities they enjoy doing), and readiness to  exercise | |  |
|  | **Contextual:** childcare (child supervision); clothing (availability of appropriate clothing and shoes); finances (problems with insurance or debt), food (access to healthy food), housing (availability of safe and secure housing), internet/broadband (availability of internet to check email and schedule exercise sessions), phone (e.g., mobile or landline; availability of a phone to connect with peer and health care professional), transportation  (availability of transportation), utilities (whether an individual had utility companies cut off service due to not paying bills), residential greenness (availability of green spaces), neighborhood safety (safety of local environment), access to a facility or exercise program in the neighborhood or at work, and access to exercise resources at home | |  |
|  | **Adverse events related to breast cancer and its treatment:** Cognitive difficulty (mental slowing), neuropathy, sarcopenia/muscle weakness,  slowing and fatigue, bone loss, bone metastases, lymphedema, symptom clusters, arthritis/musculoskeletal issues, ataxia, severe nutritional deficiencies, cardiopulmonary disease, nausea or diarrhea, sexual dysfunction, and other (open ended question) | |  |
| Tool Outputs | **Benefits associated with exercise:** Improved quality of life, more likely to stay cancer recurrence free, less likely to die of breast cancer, less likely to die of cardiovascular disease, less likely to die of all causes, improved life expectancy, less tiredness / more energy, improved cognition, greater ability to do everyday tasks, less likely to experience anxiety, less likely to experience depressive symptoms, less likely to experience lymphedema, improved bone health, improved sleep, less likely to be admitted to the hospital, less like to experience adverse side effects during treatment, and other (open ended question) | | Cancer exercise guidelines [3] and cognitive interview |

Note. NIH = National Institutes of Health; CDE = Common Data Elements.

**Table S2.** Breast cancer survivor and health care provider characteristics.

| **Characteristic** | **N** | **%** |
| --- | --- | --- |
| **Breast cancer survivor (n=26)** |  |  |
| **Education** |  |  |
| High school or less | 0 | 0.0 |
| Some college | 6 | 24.0 |
| Bachelors degree or higher | 19 | 76.0 |
| **Household income** |  |  |
| $0-$34,999 | 1 | 4.2 |
| $35,000-$74,999 | 7 | 30.4 |
| $75,000 or greater | 15 | 62.5 |
| Health care provider (n=69) |  |  |
| **Years of Experience Median (IQR)** | 69 | 7 (4-16) |
| **Type of Health care Professional** |  |  |
| Breast Oncologists (medical) | 13 | 18.8 |
| Exercise Specialists (Physiologists, Trainers, American College of Sports Medicine (ACSM)/Cancer Exercise Training Institute  /American Council on Exercise Certified) | 11 | 15.9 |
| Occupational/Physical Therapists | 12 | 17.4 |
| Patient Navigators/Social Workers/Nurses | 29 | 42.0 |
| Primary Care Physicians | 4 | 5.8 |

Note. IQR = interquartile range.

Totals may not sum to 26 for breast cancer survivors or 69 for health care providers due to missing data.

**Table S3.** Thematic analysis of respondents' open-ended responses regarding how the tool would increase their confidence to discuss exercise in clinical settings.

| **Breast Cancer Survivors**  **(n, %)** | **Health care Providers**  **(n, %)** |
| --- | --- |
| **Contribute to confidence** | |
| Provide evidence to take to provider  (n=7, 33.3%) | Access to evidence-based information  (n=16, 39.0%) |
| Knowledge to support conversation  (n=5, 23.8%) | Discussion guidance  (n=13, 31.7%) |
| Prepare for conversation  (n=4, 19.0%) | Increased health care provider knowledge (n=3, 7.3%) |
| Help initiate conversation  (n=3, 14.3%) |  |

Note. N and % refer to the number and percentage of respondents whose responses aligned with that theme.

**Table S4.** Likert scale distributions of breast cancer survivors’ and health care providers’ endorsement of usefulness, potential uses, and timing of access to the tool.

|  | | **Would increase your self-confidence to talk about exercise with your health care provider(s)/patients** | | | | **You would find useful** | | | | **You would use on a regular basis** | | | **Encourage to engage in exercise** | | | |
| --- | --- | --- | --- | --- | --- | --- | --- | --- | --- | --- | --- | --- | --- | --- | --- | --- |
| **Response** | | **Breast cancer survivors, N (%)** | | **Providers,**  **N (%)** | | **Breast cancer survivors, N (%)** | | **Providers,**  **N (%)** | | **Breast cancer survivors, N (%)** | **Providers,**  **N (%)** | | | **Breast cancer survivors, N (%)** | | **Providers,**  **N (%)** |
| Strongly disagree | | 0 (0.0) | | 3 (4.4) | | 0 (0.0) | | 2 (2.9) | | 1 (3.9) | 4 (5.8) | | | 0 (0.0) | | 0 (0.0) |
| Disagree | | 2 (7.7) | | 2 (2.9) | | 0 (0.0) | | 1 (1.5) | | 1 (3.9) | 3 (4.4) | | | 0 (0.0) | | 0 (0.0) |
| Neutral | | 2 (7.7) | | 5 (7.3) | | 3 (11.5) | | 4 (5.8) | | 4 (15.4) | 9 (13.0) | | | 0 (0.0) | | 2 (2.9) |
| Agree | | 5 (19.2) | | 22 (31.9) | | 8 (30.8) | | 17 (24.6) | | 6 (23.1) | 22 (31.9) | | | 1 (3.9) | | 25 (36.2) |
| Strongly agree | | 17 (65.4) | | 37 (53.6) | | 15 (57.7) | | 45 (65.2) | | 14 (53.9) | 31 (44.9) | | | 22 (84.6) | | 35 (50.7) |
| Mean (SD)^a^ | | 4.42 (0.95) | | 4.28 (1.03) | | 4.46 (0.71) | | 4.48 (0.90) | | 4.19 (1.10) | 4.06 (1.14) | | | 4.96 (0.21) | | 4.53 (0.56) |
| Total, N | | 26 | | 69 | | 26 | | 69 | | 26 | 69 | | | 23 | | 62 |
|  | **Educate on the benefits (and harms) of engaging in exercise** | | | | **Refer to exercise professionals** | | | | | **Facilitate shared decision-making between patient and provider about engaging in exercise** | | **Identify resources available within the health care system to support engagement in exercise** | | | | |
| **Response** | **Breast cancer survivors, N (%)** | | **Providers,**  **N (%)** | | | | **Breast cancer survivors, N (%)** | | **Providers,**  **N (%)** | **Breast cancer survivors, N (%)** | **Providers,**  **N (%)** | | **Breast cancer survivors, N (%)** | | **Providers,**  **N (%)** | |
| Strongly disagree | 1 (3.9) | | 0 (0.0) | | | | 0 (0.0) | | 0 (0.0) | 1 (3.9) | 0 (0.0) | | 0 (0.0) | | 0 (0.0) | |
| Disagree | 1 (3.9) | | 0 (0.0) | | | | 1 (3.9) | | 1 (1.5) | 1 (3.9) | 0 (0.0) | | 1 (3.9) | | 1 (1.5) | |
| Neutral | 4 (15.4) | | 4 (5.8) | | | | 1 (3.9) | | 9 (13.0) | 0 (0.0) | 5 (7.3) | | 1 (3.9) | | 6 (8.7) | |
| Agree | 17 (65.4) | | 23 (33.3) | | | | 9 (34.6) | | 20 (29.0) | 7 (26.9) | 25 (36.2) | | 4 (15.4) | | 20 (29.0) | |
| Strongly agree | 3 (11.5) | | 35 (50.7) | | | | 11 (42.3) | | 31 (44.9) | 14 (53.9) | 32 (46.4) | | 17 (65.4) | | 35 (50.7) | |
| Mean (SD)^a^ | 4.57 (0.95) | | 4.50 (0.62) | | | | 4.36 (0.79) | | 4.33 (0.79) | 4.39 (1.03) | 4.44 (0.64) | | 4.61 (0.78) | | 4.44 (0.74) | |
| Total, N | 23 | | 62 | | | | 22 | | 61 | 23 | 62 | | 23 | | 62 | |
|  | **Before a medical appointment (individually)** | | | | | | **Before a medical appointment (with a care coordinator or patient navigator)** | | | **During a medical appointment with a health care provider** | | | **After a medical appointment (individually)** | | | |
| **Response** | **Breast cancer survivors, N (%)** | | **Providers,**  **N (%)** | | | | **Breast cancer survivors, N (%)** | | **Providers,**  **N (%)** | **Breast cancer survivors, N (%)** | **Providers,**  **N (%)** | | **Breast cancer survivors, N (%)** | | **Providers,**  **N (%)** | |
| Strongly disagree | 2 (7.7) | | 2 (2.9) | | | | 1 (3.9) | | 1 (1.5) | 1 (3.9) | 1 (1.5) | | 1 (3.9) | | 0 (0.0) | |
| Disagree | 1 (3.9) | | 12 (17.4) | | | | 3 (11.5) | | 12 (17.4) | 1 (3.9) | 5 (7.3) | | 3 (11.5) | | 2 (2.9) | |
| Neutral | 4 (15.4) | | 12 (17.4) | | | | 5 (19.2) | | 14 (20.3) | 4 (15.4) | 7 (10.1) | | 4 (15.4) | | 7 (10.1) | |
| Agree | 9 (34.6) | | 30 (43.5) | | | | 9 (34.6) | | 24 (34.8) | 9 (34.6) | 28 (40.6) | | 9 (34.6) | | 28 (40.6) | |
| Strongly agree | 10 (38.5) | | 13 (18.8) | | | | 8 (30.8) | | 17 (24.6) | 11 (42.3) | 27 (39.1) | | 9 (34.6) | | 29 (42.0) | |
| Mean (SD)^a^ | 3.92 (1.20) | | 3.58 (1.08) | | | | 3.77 (1.14) | | 3.65 (1.09) | 4.08 (1.06) | 4.10 (0.96) | | 3.85 (1.16) | | 4.27 (0.78) | |
| Total, N | 26 | | 69 | | | | 26 | | 68 | 26 | 68 | | 26 | | 66 | |
|  | | **After a medical appointment (with a care coordinator or patient navigator)** | | | |  |  |  |  |  |  |  |  |  |  |  |
| **Response** | | **Breast cancer survivors, N (%)** | | **Providers,**  **N (%)** | |  |  |  |  |  |  |  |  |  |  |  |
| Strongly disagree | | 1 (3.9) | | 0 (0.0) | |  |  |  |  |  |  |  |  |  |  |  |
| Disagree | | 0 (0.0) | | 2 (2.9) | |  |  |  |  |  |  |  |  |  |  |  |
| Neutral | | 5 (19.2) | | 7 (10.1) | |  |  |  |  |  |  |  |  |  |  |  |
| Agree | | 9 (34.6) | | 20 (29.0) | |  |  |  |  |  |  |  |  |  |  |  |
| Strongly agree | | 11 (42.3) | | 38 (55.1) | |  |  |  |  |  |  |  |  |  |  |  |
| Mean (SD)^a^ | | 4.12 (0.99) | | 4.40 (0.80) | |  |  |  |  |  |  |  |  |  |  |  |
| Total, N | | 26 | | 67 | |  |  |  |  |  |  |  |  |  |  |  |

Note. SD = standard deviation.

^a^Based on 5‑point Likert scale (1 = Strongly disagree, 5 = Strongly agree).

**Table S5.** Survey items with the highest endorsement among breast cancer survivors and health care providers. Responses on the 5-point Likert scale (strongly disagree to strongly agree) were dichotomized as endorsement (agree/strongly agree) vs nonendorsement.

| **Breast cancer survivor** | | | | **Healthcare provider** | | | |
| --- | --- | --- | --- | --- | --- | --- | --- |
| **Survey item(s)** | **N** | **%^a^** | **95% CI** | **Survey item(s)** | **N** | **%^a^** | **95% CI** |
| **Demographic and clinical (breast cancer-related)** | | | | | | | |
| Current treatment | 26 | 84.6 | 65.1–95.6 | Current treatment | 69 | 94.2 | 85.8–98.4 |
| Historical treatment / Stage at diagnosis | 26 | 80.8 | 60.6–93.4 | Age | 69 | 89.9 | 80.2–95.8 |
| Tumor characteristics (e.g., tumor grade) | 26 | 76.9 | 56.4–91.0 | Historical treatment | 69 | 82.6 | 71.6–90.7 |
| **Overall health and exercise** | | | | | | | |
| Readiness to exercise | 26 | 84.6 | 65.1–95.6 | Functional / Physical / Cognitive impairment | 69 | 100 | 94.8–100 |
| Current comorbidities | 25 | 80.0 | 59.3–93.2 | Patient personal activity preferences | 69 | 98.6 | 92.2–100 |
| Cognitive impairment | 26 | 76.9 | 56.4–91.0 | Readiness to exercise | 69 | 95.7 | 87.8–99.1 |
| **Contextual** | | | | | | | |
| Access to exercise resources at home | 26 | 92.3 | 74.9–99.1 | Access to exercise resources at home | 69 | 92.8 | 83.9–97.6 |
| Access to healthy food | 26 | 84.6 | 65.1–95.6 | Availability of safe and secure housing) / Childcare | 69 | 89.9 | 80.2–95.8 |
| Access to a local facility or exercise program | 26 | 80.8 | 60.6–93.4 | Access to healthy food / Phone | 69 | 87.0 | 76.7–93.9 |
| **Benefits** | | | | | | | |
| Lower treatment side effects / cardiovascular death risk | 26 | 92.3 | 74.9–99.1 | Improved quality of life | 69 | 100 | 94.8–100 |
| Improved life expectancy | 25 | 92.0 | 74.0–99.0 | Improved bone health / Greater ability to do everyday tasks | 68 | 98.5 | 92.1–100 |
| Improved bone health / quality of life | 26 | 88.5 | 69.8–97.6 | Lower depressive symptoms | 69 | 97.1 | 89.9–99.6 |
| **Adverse events related to breast cancer and its treatment** | | | | | | | |
| Slowing and fatigue / Sarcopenia / Neuropathy | 26 | 92.3 | 74.9–99.1 | Slowing and fatigue | 67 | 98.5 | 92.0–100 |
| Ataxia | 26 | 88.5 | 69.8–97.6 | Lymphedema | 69 | 97.1 | 89.9–99.6 |
| Lymphedema | 25 | 88.0 | 68.8–97.5 | Bone loss | 69 | 95.7 | 87.8–99.1 |

**Table S6.** Breast cancer survivors’ and health care providers’ endorsement of inputs and outputs for inclusion in an exercise clinical decision tool.

|  | **Breast cancer survivor** | | **95% CI** | |  |  | **Health care provider** | | **95% CI** | |
| --- | --- | --- | --- | --- | --- | --- | --- | --- | --- | --- |
| **Survey item** | **N** | **%^a^** | **Lower** | **Upper** |  | **Survey item** | **N** | **%^a^** | **Lower** | **Upper** |
| **Demographic and clinical (breast cancer-related)** | | | | | | | | | | |
| Current treatment(s) | 26 | 84.6% | 65.1% | 95.6% |  | Current treatment(s) | 69 | 94.2% | 85.8% | 98.4% |
| Historical treatments(s) | 26 | 80.8% | 60.6% | 93.4% |  | Historical treatments(s) | 69 | 82.6% | 71.6% | 90.7% |
| Stage at diagnosis  (e.g., Stage I/localized) | 26 | 80.8% | 60.6% | 93.4% |  | Stage at diagnosis  (e.g., Stage I/localized) | 69 | 82.6% | 71.6% | 90.7% |
| Tumor characteristics  (e.g., tumor grade) | 26 | 76.9% | 56.4% | 91.0% |  | Tumor characteristics  (e.g., tumor grade) | 68 | 63.2% | 50.7% | 74.6% |
| Age | 26 | 73.1% | 52.2% | 88.4% |  | Age | 69 | 89.9% | 80.2% | 95.8% |
| Race and ethnicity | 25 | 40.0% | 21.1% | 61.3% |  | Race and ethnicity | 69 | 58.0% | 45.5% | 69.8% |
| **Overall health and exercise** | | | | | | | | | | |
| Readiness to exercise | 26 | 84.6% | 65.1% | 95.6% |  | Readiness to exercise | 69 | 95.7% | 87.8% | 99.1% |
| Current comorbidities | 25 | 80.0% | 59.3% | 93.2% |  | Current comorbidities | 68 | 95.6% | 87.6% | 99.1% |
| Cognitive impairment | 26 | 76.9% | 56.4% | 91.0% |  | Cognitive impairment | 69 | 100.0% | 94.8% | 100.0% |
| Physical impairments | 26 | 73.1% | 52.2% | 88.4% |  | Physical impairments | 69 | 100.0% | 94.8% | 100.0% |
| Functional impairments | 26 | 73.1% | 52.2% | 88.4% |  | Functional impairments | 69 | 100.0% | 94.8% | 100.0% |
| Psychological impairment | 26 | 65.4% | 44.3% | 82.8% |  | Psychological impairment | 69 | 97.1% | 89.9% | 99.6% |
| Patient personal  preferences | 26 | 61.5% | 40.6% | 79.8% |  | Patient personal preferences | 69 | 98.6% | 92.2% | 100.0% |
| **Contextual** |  |  |  |  |  |  |  |  |  |  |
| Access to exercise  resources at home | 26 | 92.3% | 74.9% | 99.1% |  | Access to exercise resources  at home | 69 | 92.8% | 83.9% | 97.6% |
| Access to healthy food | 26 | 84.6% | 65.1% | 95.6% |  | Childcare | 69 | 89.9% | 80.2% | 95.8% |
| Access to a local exercise  facility or program | 26 | 80.8% | 60.6% | 93.4% |  | Housing | 69 | 89.9% | 80.2% | 95.8% |
| Neighborhood safety | 26 | 69.2% | 48.2% | 85.7% |  | Access to healthy food | 69 | 87.0% | 76.7% | 93.9% |
| Financial problems | 26 | 65.4% | 44.3% | 82.8% |  | Phone | 69 | 87.0% | 76.7% | 93.9% |
| Childcare | 26 | 61.5% | 40.6% | 79.8% |  | Access to a local exercise  facility or program | 69 | 85.5% | 75.0% | 92.8% |
| Availability of appropriate  clothing | 26 | 57.7% | 36.9% | 76.6% |  | Financial problems | 69 | 85.5% | 75.0% | 92.8% |
| Housing | 26 | 53.8% | 33.4% | 73.4% |  | Neighborhood safety | 69 | 84.1% | 73.3% | 91.8% |
| Transportation | 26 | 53.8% | 33.4% | 73.4% |  | Transportation | 68 | 83.8% | 72.9% | 91.6% |
| Internet/Broadband | 26 | 50.0% | 29.9% | 70.1% |  | Internet/Broadband | 69 | 81.2% | 69.9% | 89.6% |
| Phone | 26 | 50.0% | 29.9% | 70.1% |  | Utilities cut off service due to  not paying bills | 69 | 78.3% | 66.7% | 87.3% |
| Residential greenness | 26 | 42.3% | 23.4% | 63.1% |  | Residential greenness | 69 | 65.2% | 52.8% | 76.3% |
| Utilities cut off service due  to not paying bills | 26 | 38.5% | 20.2% | 59.4% |  | Availability of appropriate  clothing | 69 | 59.4% | 46.9% | 71.1% |
| **Benefits** |  |  |  |  |  | |  |  |  |  |
| Lower cardiovascular  death risk | 26 | 92.3% | 74.9% | 99.1% |  | Improved quality of life | 69 | 100.0% | 94.8% | 100.0% |
| Lower treatment side  effects | 26 | 92.3% | 74.9% | 99.1% |  | Improved bone health | 68 | 98.5% | 92.1% | 100.0% |
| Improved life expectancy | 25 | 92.0% | 74.0% | 99.0% |  | Greater ability to do everyday  tasks | 68 | 98.5% | 92.1% | 100.0% |
| Improved bone health | 26 | 88.5% | 69.8% | 97.6% |  | Lower depressive symptoms | 69 | 97.1% | 89.9% | 99.6% |
| Improved quality of life | 26 | 88.5% | 69.8% | 97.6% |  | Improved sleep | 69 | 95.7% | 87.8% | 99.1% |
| Greater ability to do  everyday tasks | 26 | 84.6% | 65.1% | 95.6% |  | Lower anxiety | 68 | 95.6% | 87.6% | 99.1% |
| Lower anxiety | 26 | 84.6% | 65.1% | 95.6% |  | Lower treatment side effects | 69 | 94.2% | 85.8% | 98.4% |
| More energy | 26 | 84.6% | 65.1% | 95.6% |  | More energy | 69 | 94.2% | 85.8% | 98.4% |
| Improved sleep | 26 | 80.8% | 60.6% | 93.4% |  | Lower lymphedema | 69 | 94.2% | 85.8% | 98.4% |
| Lower hospital admission | 26 | 80.8% | 60.6% | 93.4% |  | Improved cognition (memory) | 68 | 94.1% | 85.6% | 98.4% |
| Lower recurrence risk | 26 | 80.8% | 60.6% | 93.4% |  | Improved life expectancy | 69 | 89.9% | 80.2% | 95.8% |
| Improved cognition  (memory) | 26 | 76.9% | 56.4% | 91.0% |  | Lower cardiovascular death  risk | 69 | 88.4% | 78.4% | 94.9% |
| Lower breast cancer  death risk | 26 | 76.9% | 56.4% | 91.0% |  | Lower hospital admission | 69 | 87.0% | 76.7% | 93.9% |
| Lower depressive  symptoms | 26 | 73.1% | 52.2% | 88.4% |  | Lower recurrence risk | 69 | 87.0% | 76.7% | 93.9% |
| Lower all-cause death risk | 26 | 69.2% | 48.2% | 85.7% |  | Lower breast cancer death risk | 69 | 87.0% | 76.7% | 93.9% |
| Lower lymphedema | 26 | 69.2% | 48.2% | 85.7% |  | Lower all-cause death risk | 68 | 80.9% | 69.5% | 89.4% |
| **Adverse events related to breast cancer and its treatment** | | | | | | | | | | |
| Neuropathy | 26 | 92.3% | 74.9% | 99.1% |  | Slowing and fatigue | 67 | 98.5% | 92.0% | 100.0% |
| Sarcopenia | 26 | 92.3% | 74.9% | 99.1% |  | Lymphedema | 69 | 97.1% | 89.9% | 99.6% |
| Slowing and fatigue | 26 | 92.3% | 74.9% | 99.1% |  | Sarcopenia | 69 | 95.7% | 87.8% | 99.1% |
| Ataxia | 26 | 88.5% | 69.8% | 97.6% |  | Bone loss | 69 | 95.7% | 87.8% | 99.1% |
| Lymphedema | 25 | 88.0% | 68.8% | 97.5% |  | Neuropathy | 69 | 92.8% | 83.9% | 97.6% |
| Symptom clusters | 26 | 84.6% | 65.1% | 95.6% |  | Ataxia | 69 | 92.8% | 83.9% | 97.6% |
| Arthritis/musculoskeletal  issues | 26 | 80.8% | 60.6% | 93.4% |  | Bone metastasis | 69 | 91.3% | 82.0% | 96.7% |
| Cognitive difficulty (mental  slowing) | 26 | 80.8% | 60.6% | 93.4% |  | Cardiopulmonary disease | 69 | 88.4% | 78.4% | 94.9% |
| Severe nutritional  deficiencies | 26 | 80.8% | 60.6% | 93.4% |  | Arthritis/musculoskeletal  issues | 67 | 88.1% | 77.8% | 94.7% |
| Bone loss | 25 | 80.0% | 59.3% | 93.2% |  | Cognitive difficulty (mental  slowing) | 69 | 87.0% | 76.7% | 93.9% |
| Bone metastasis | 26 | 76.9% | 56.4% | 91.0% |  | Sexual dysfunction | 69 | 87.0% | 76.7% | 93.9% |
| Cardiopulmonary disease | 26 | 73.1% | 52.2% | 88.4% |  | Symptom clusters | 69 | 84.1% | 73.3% | 91.8% |
| Nausea or diarrhea | 26 | 69.2% | 48.2% | 85.7% |  | Nausea or diarrhea | 69 | 81.2% | 69.9% | 89.6% |
| Sexual dysfunction | 26 | 65.4% | 44.3% | 82.8% |  | Severe nutritional deficiencies | 69 | 79.7% | 68.3% | 88.4% |

Note. CI=Confidence interval.

^a^Likert-scale responses (1 = strongly disagree to 5 = strongly agree) were dichotomized as endorsement (agree/strongly agree) versus non-endorsement (neutral or disagree). Percentages reflect within-group endorsement rates.

**Table S7.** Mean (SD) of Likert scale (1=strongly disagree to 5=strongly agree) responses among breast cancer survivors and health care providers.

| **Survey item** | **N** | **Mean** | **SD** |  | **Survey item** | **N** | **Mean** | **SD** |
| --- | --- | --- | --- | --- | --- | --- | --- | --- |
| **Demographic and clinical (breast cancer-related)** | | | | | | | | |
| Current treatment(s) | 26 | 4.42 | 1.06 |  | Current treatment(s) | 69 | 4.62 | 0.71 |
| Stage at diagnosis (e.g., Stage I/localized) | 26 | 4.23 | 1.07 |  | Age | 69 | 4.45 | 0.78 |
| Historical treatments(s) | 26 | 4.15 | 1.05 |  | Historical treatments(s) | 69 | 4.25 | 0.88 |
| Tumor characteristics (e.g., tumor grade) | 26 | 4.15 | 1.08 |  | Stage at diagnosis (e.g., Stage I/localized) | 69 | 4.23 | 0.84 |
| Age | 26 | 3.88 | 1.34 |  | Tumor characteristics (e.g., tumor grade) | 68 | 3.74 | 1.10 |
| Race and ethnicity | 25 | 3.00 | 1.32 |  | Race and ethnicity | 69 | 3.39 | 1.29 |
| **Overall health and exercise** |  |  |  |  |  |  |  |  |
| Readiness to exercise | 26 | 4.35 | 0.85 |  | Physical impairments | 69 | 4.86 | 0.35 |
| Current comorbidities | 25 | 4.24 | 1.16 |  | Functional impairments | 69 | 4.81 | 0.39 |
| Physical impairments | 26 | 4.19 | 1.27 |  | Current comorbidities | 68 | 4.78 | 0.51 |
| Cognitive impairment | 26 | 4.04 | 1.28 |  | Cognitive impairment | 69 | 4.74 | 0.44 |
| Patient personal preferences | 26 | 3.96 | 1.11 |  | Patient personal preferences | 69 | 4.72 | 0.54 |
| Functional impairments | 26 | 3.92 | 1.26 |  | Readiness to exercise | 69 | 4.58 | 0.58 |
| Psychological impairment | 26 | 3.81 | 1.33 |  | Psychological impairment | 69 | 4.52 | 0.61 |
| **Contextual** |  |  |  |  |  |  |  |  |
| Access to exercise resources at home | 26 | 4.38 | 0.90 |  | Access to exercise resources at home | 69 | 4.43 | 0.72 |
| Access to healthy food | 26 | 4.35 | 0.85 |  | Housing | 69 | 4.41 | 0.79 |
| Access to a local exercise facility or  program | 26 | 4.08 | 0.80 |  | Access to healthy food | 69 | 4.39 | 0.79 |
| Financial problems | 26 | 3.88 | 1.03 |  | Transportation | 68 | 4.31 | 0.89 |
| Neighborhood safety | 26 | 3.85 | 0.88 |  | Neighborhood safety | 69 | 4.28 | 0.80 |
| Childcare | 26 | 3.58 | 1.03 |  | Childcare | 69 | 4.26 | 0.76 |
| Housing | 26 | 3.58 | 1.03 |  | Financial problems | 69 | 4.20 | 0.88 |
| Phone | 26 | 3.58 | 1.14 |  | Phone | 69 | 4.19 | 0.69 |
| Availability of appropriate clothing | 26 | 3.50 | 0.81 |  | Access to a local exercise facility or  program | 69 | 4.17 | 0.75 |
| Transportation | 26 | 3.50 | 1.24 |  | Utilities cut off service due to not paying  bills | 69 | 4.10 | 0.81 |
| Internet/Broadband | 26 | 3.46 | 1.10 |  | Internet/Broadband | 69 | 4.01 | 0.76 |
| Residential greenness | 26 | 3.23 | 1.03 |  | Residential greenness | 69 | 3.70 | 0.91 |
| Utilities cut off service due to not paying  bills | 26 | 3.19 | 1.13 |  | Availability of appropriate clothing | 69 | 3.70 | 0.96 |
| **Benefits** |  |  |  |  |  |  |  |  |
| Lower treatment side effects | 26 | 4.58 | 0.64 |  | Improved quality of life | 69 | 4.78 | 0.42 |
| Improved quality of life | 26 | 4.58 | 0.90 |  | Greater ability to do everyday tasks | 68 | 4.74 | 0.48 |
| Improved life expectancy | 25 | 4.56 | 0.77 |  | Lower depressive symptoms | 69 | 4.67 | 0.53 |
| More energy | 26 | 4.54 | 0.76 |  | More energy | 69 | 4.67 | 0.59 |
| Lower cardiovascular death risk | 26 | 4.42 | 0.64 |  | Improved sleep | 69 | 4.62 | 0.57 |
| Improved sleep | 26 | 4.38 | 0.90 |  | Lower treatment side effects | 69 | 4.61 | 0.75 |
| Improved bone health | 26 | 4.35 | 0.94 |  | Improved life expectancy | 69 | 4.59 | 0.71 |
| Greater ability to do everyday tasks | 26 | 4.31 | 0.97 |  | Improved cognition (memory) | 68 | 4.59 | 0.60 |
| Lower recurrence risk | 26 | 4.27 | 1.08 |  | Lower anxiety | 68 | 4.56 | 0.58 |
| Lower anxiety | 26 | 4.23 | 1.11 |  | Lower lymphedema | 69 | 4.54 | 0.61 |
| Improved cognition (memory) | 26 | 4.23 | 0.91 |  | Lower breast cancer death risk | 69 | 4.54 | 0.85 |
| Lower breast cancer death risk | 26 | 4.19 | 1.27 |  | Lower recurrence risk | 69 | 4.51 | 0.83 |
| Lower hospital admission | 26 | 4.12 | 1.21 |  | Improved bone health | 68 | 4.50 | 0.53 |
| Lower depressive symptoms | 26 | 4.04 | 1.31 |  | Lower cardiovascular death risk | 69 | 4.43 | 0.70 |
| Lower all-cause death risk | 26 | 3.85 | 1.26 |  | Lower hospital admission | 69 | 4.42 | 0.76 |
| Lower lymphedema | 26 | 3.81 | 1.39 |  | Lower all-cause death risk | 68 | 4.28 | 0.90 |
|  |  |  |  |  |  |  |  |  |
| **Adverse events related to breast cancer and its treatment** | | | | | | | | |
| Sarcopenia | 26 | 4.62 | 0.75 |  | Slowing and fatigue | 67 | 4.61 | 0.52 |
| Slowing and fatigue | 26 | 4.58 | 0.90 |  | Lymphedema | 69 | 4.61 | 0.55 |
| Neuropathy | 26 | 4.54 | 0.76 |  | Sarcopenia | 69 | 4.55 | 0.58 |
| Lymphedema | 25 | 4.40 | 1.12 |  | Neuropathy | 69 | 4.55 | 0.63 |
| Ataxia | 26 | 4.35 | 1.13 |  | Bone loss | 69 | 4.51 | 0.58 |
| Bone loss | 25 | 4.32 | 0.90 |  | Ataxia | 69 | 4.51 | 0.63 |
| Bone metastasis | 26 | 4.27 | 1.08 |  | Bone metastasis | 69 | 4.51 | 0.66 |
| Symptom clusters | 26 | 4.12 | 1.18 |  | Cardiopulmonary disease | 69 | 4.45 | 0.70 |
| Cognitive difficulty (mental slowing) | 26 | 4.08 | 1.02 |  | Cognitive difficulty (mental slowing) | 69 | 4.33 | 0.78 |
| Severe nutritional deficiencies | 26 | 4.08 | 1.13 |  | Arthritis/musculoskeletal issues | 67 | 4.31 | 0.72 |
| Arthritis/musculoskeletal issues | 26 | 4.04 | 1.18 |  | Severe nutritional deficiencies | 69 | 4.23 | 0.84 |
| Cardiopulmonary disease | 26 | 4.04 | 1.25 |  | Nausea or diarrhea | 69 | 4.22 | 0.74 |
| Nausea or diarrhea | 26 | 3.92 | 1.13 |  | Symptom clusters | 69 | 4.16 | 0.72 |
| Sexual dysfunction | 26 | 3.77 | 1.14 |  | Sexual dysfunction | 68 | 3.96 | 0.95 |

Note. SD = standard deviation.

**
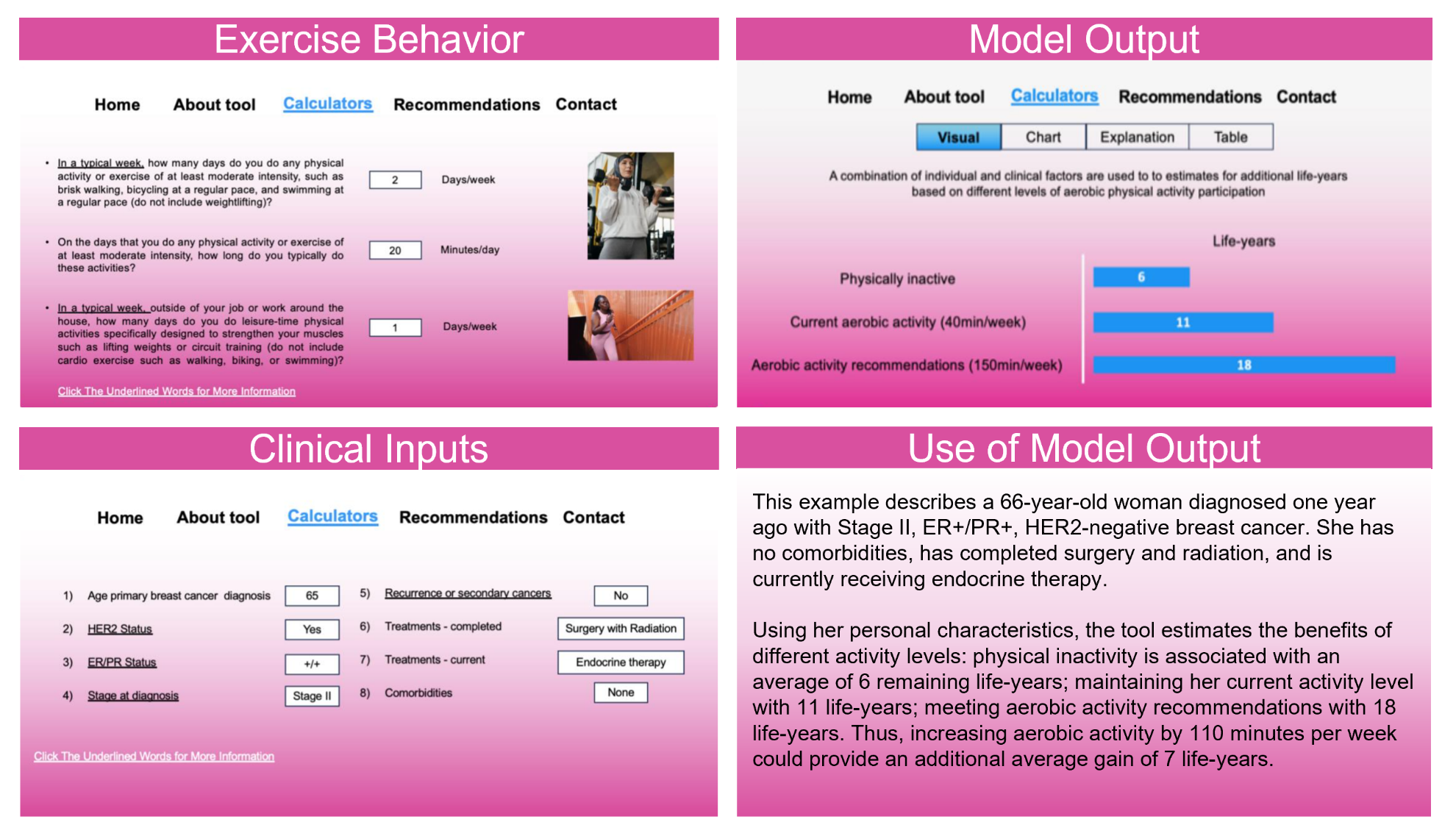
**

**Figure S1.** Screenshot of the paper draft of the prototype clinical decision tool for individualized exercise discussions shown to study participants.


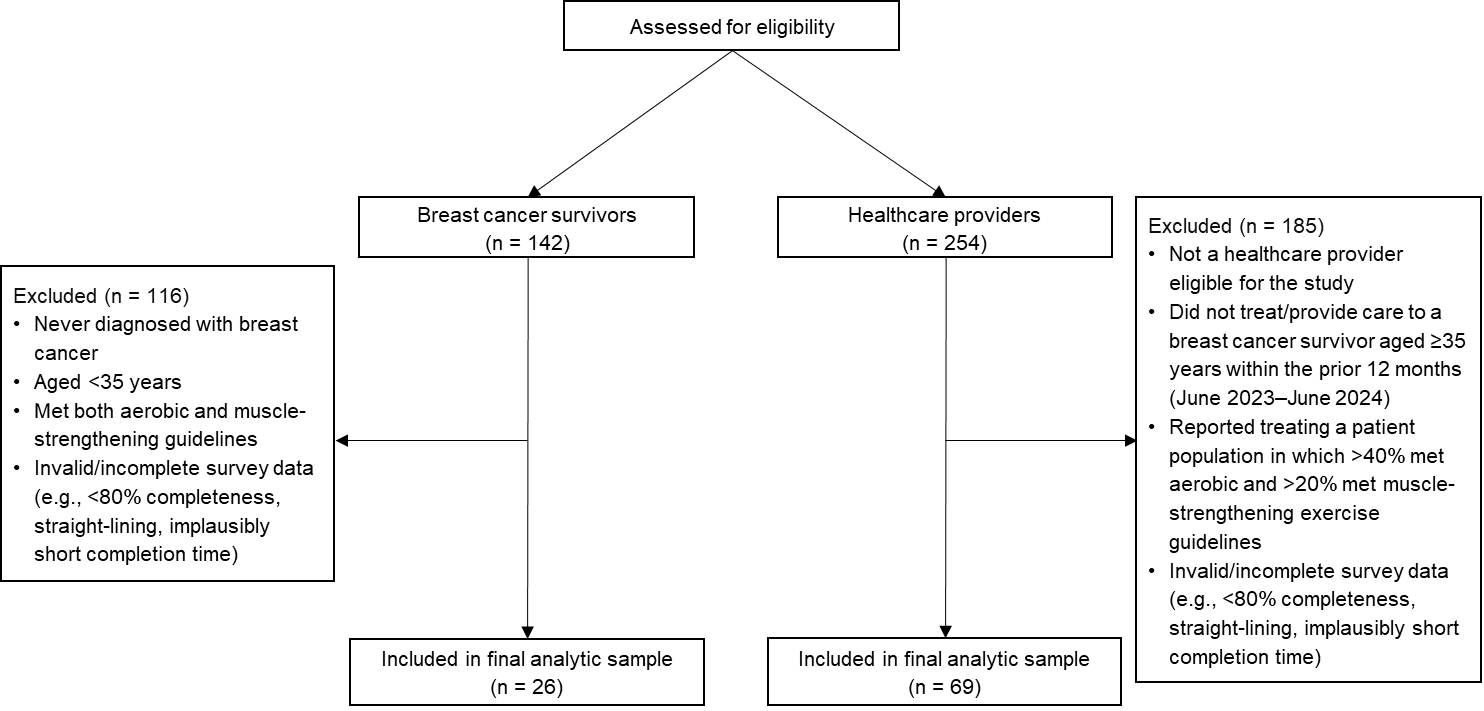


**Figure S2.** Flow diagram of study participants (breast cancer survivors and health care providers).

## References

1. National Library of Medicine. NIH CDE Repository. 2024.
2. Powers SL, Pitas NA, Mowen AJ. Critical consciousness of systemic racism in parks among park agency directors and policymakers: An environmental justice tool for recreation and conservation leaders. *Soc Nat Resour*. 2023:1-24. doi: 10.1080/08941920.2023.2250737.
3. Campbell KL, Winters-Stone KM, Wiskemann J, May AM, Schwartz AL, Courneya KS, et al. Exercise guidelines for cancer survivors: Consensus statement from international multidisciplinary roundtable. *Med Sci Sports Exerc*. 2019;51(11):2375-90. PMID: 31626055. doi: 10.1249/mss.0000000000002116.
4. Nadler MB, Bainbridge D, Fong AJ, Sussman J, Tomasone JR, Neil-Sztramko SE. Moving Cancer Care Ontario's Exercise for People with Cancer guidelines into oncology practice: using the Theoretical Domains Framework to validate a questionnaire. *Support Care Cancer*. 2019 Jun;27(6):1965-8. PMID: 30762143. doi: 10.1007/s00520-019-04689-1.
5. O'Neill SC, Taylor KL, Clapp J, Jayasekera J, Isaacs C, Mary Atieh Graham D, et al. Multilevel influences on patient-oncologist communication about genomic test results: Oncologist perspectives. *J Health Commun*. 2018;23(7):679-86. PMID: 30130477. doi: 10.1080/10810730.2018.1506836.
